# Prevalence and patterns of physical activity, sedentary behaviour, and their association with health-related quality of life within a multi-ethnic Asian population

**DOI:** 10.1186/s12889-021-11902-6

**Published:** 2021-10-25

**Authors:** Jue Hua Lau, Asharani Nair, Edimansyah Abdin, Roystonn Kumarasan, Peizhi Wang, Fiona Devi, Chee Fang Sum, Eng Sing Lee, Falk Müller-Riemenschneider, Mythily Subramaniam

**Affiliations:** 1 Present Address: Research Division, Institute of Mental Health, Buangkok Green Medical Park, 10 Buangkok View, Singapore, 539747 Singapore; 2 Admiralty Medical Centre and Khoo Teck Puat Hospital, Singapore, Singapore; 3 National Healthcare Group Polyclinics, Singapore, Singapore; 4 Saw Swee Hock School of Public Health and Yong Loo Lin School of Medicine, National University of Singapore, Singapore, Singapore

**Keywords:** Physical activity, Sedentary behaviour, GPAQ, Singapore

## Abstract

**Objective:**

The study aimed to examine the prevalence and sociodemographic correlates of physical activity and sedentary behaviour in the general population of the multi-ethnic nation of Singapore as part of the Knowledge, Practice and Attitudes towards Diabetes study, a cross-sectional and population-based survey. It also examined the relationship between physical activity, sedentary behaviour, and health-related quality of life (HRQoL).

**Methods:**

Physical activity and sedentary behaviour were assessed via the Global Physical Activity Questionnaire (GPAQ), while physical and mental HRQoL was assessed via the Short Form Health Survey (SF-12v2). Survey weights were employed to account for complex survey design. Multivariable logistic regression models were utilized to examine sociodemographic correlates of physical activity (insufficient vs. sufficient physical activity) and sedentary behaviour (< 7 h/day vs ≥7 h/day). Descriptive statistics were calculated to examine the percentage of time spent in different domains of physical activity. Multivariable linear regressions were conducted to examine the association between physical activity and sedentary behaviour with physical and mental HRQoL.

**Results:**

Two thousand eight hundred sixty seven participants recruited from February 2019 to March 2020 (prior to COVID-19 lockdown and related restrictions in Singapore) were included in the analyses. 83.3% of respondents had sufficient physical activity. Age (65 years and above) and income (SGD 2000 to 3999) were associated with a higher likelihood of insufficient physical activity. In contrast, those of Malay ethnicity and having one chronic physical condition were associated with a lower likelihood of insufficient physical activity. 47.7% reported that they had sedentary behaviour of ≥7 h/day. Older age and a primary school education were related to a lower likelihood of sedentary behaviour, while being single, having higher income, obesity, and multimorbidity were associated with higher sedentary behaviour. Insufficient physical activity was significantly associated with lower physical HRQoL but was not significantly associated with mental HRQoL. Sedentary behaviour was not significantly associated with mental or physical HRQoL.

**Conclusion:**

About 17% of the population did not meet the minimum requirements for physical activity, while around half of the population spent a considerable time being sedentary. As insufficient physical activity was associated with poorer physical HRQoL, policymakers should promote moderate physical activity and encouraging the breaking up of prolonged sedentary periods within the middle- and high-income groups, especially at the workplace. Increased leisure-time exercise should be encouraged for those in the lower- income group.

**Supplementary Information:**

The online version contains supplementary material available at 10.1186/s12889-021-11902-6.

## Background

Regular physical activity confers benefits for multiple health outcomes such as reduction in all-cause mortality, non-communicable diseases, symptoms of anxiety and depression, and improved mental health [74]. Singapore, a multi-ethnic nation- state located in Southeast Asia, has launched several campaigns aimed at encouraging its citizens to exercise and live healthily. This is in part due to a rise in non-communicable conditions such as hyperlipidaemia, hypertension, diabetes, and obesity [29]. Reports from cross-sectional and population-wide studies estimated that 60.9% of the population had sufficient physical activity in 2010 [49], 73.8% in 2013 [71], and approximately 80% in 2017 and 2019 (Health Promotion [31]).

Increasing evidence in the last decade has linked sedentary behaviour with adverse health outcomes, including a higher risk of chronic physical conditions such as diabetes and cardiovascular conditions [74]. There is evidence that the direct effect of sedentary behaviour on health appears to be independent of physical activity [21, 38]. Reports of sedentary behaviour within the Singapore population are sparse and not directly comparable due to the utilization of different thresholds. For example, Sloan et al. [63] split their data on sedentary behaviour in 2010 by tertiles, with 39.2% of the study population reporting sedentary hours of ≥5 h/day while another 32.6% reporting ≥10 h/day. The latest nationwide prevalence of sedentary behaviour at the time of writing was based on data collected in 2013, with Win et al. [71] reporting that 37% of Singaporeans had ≥8 h/day of self-reported sedentary behaviour. There was thus a need for more up- to- date data on the Singapore population.

Health-related Quality of Life (HRQoL) is a construct that covers physical, psychological, and social health and represents the overall health of an individual [64]. The assessment of HRQoL via standardized instruments such as the Short Form Health Survey (SF-12v2) is necessary as it monitors the population’s health status over time, identifies health differences between certain groups, and provides information for the planning, implementation, and evaluation of health interventions [10]. While some researchers have found that physical activity was positively associated with HRQoL [6, 27, 40, 46, 55] other studies have reported that high sedentary behaviour was negatively associated with HRQoL [30, 65]. Understanding how physical activity and sedentary behaviour are associated with HRQoL within the Singapore population may help to identify groups of at-risk individuals to promote behavioural change.

To address the current gaps in the literature, the aims of the present study are threefold: i) to establish more updated nationwide data on prevalence of physical activity and sedentary behaviour within the Singapore population, ii) identify sociodemographic correlates that are related to low physical activity and high sedentary behaviour, and iii) to examine how physical activity and sedentary behaviour influence physical and mental HRQoL. The study hopes to identify specific subgroups of the population that can be engaged and encouraged to increase their physical activity through additional resource allocation and culturally appropriate interventions.

## Methods

### Sample and procedure

The present paper was part of a population- based, cross-sectional study aimed at evaluating the Knowledge, Practice, and Attitudes (KAP [3]) towards Diabetes Mellitus (DM) amongst residents of Singapore aged 18 years and above. The sample was randomly selected via a disproportionate stratified sampling design according to the ethnicity (Chinese, Malay, Indian, Others) and age groups (18–34 years, 35–49 years, 50–64 years, 65 years and above) from a national population registry database of all citizens and permanent residents within Singapore. The study oversampled certain minority populations, such as Malay and Indian ethnicity, as well as those above 65 years of age, in order to ensure sufficient sample size and to improve the reliability of the parameter estimates for these subgroups.

Citizens and permanent residents who were selected were sent notification letters followed by home visits by a trained interviewer from a survey research company to obtain their agreement and informed consent to participate in the study. Face-to-face interviews with those who agreed to participate were conducted in their preferred language (English, Mandarin, Malay, or Tamil). Responses were captured using computer- assisted personal interviewing. Individuals who were unable to be contacted due to incomplete or incorrect addresses, were living outside of the country, or were incapable of doing the interview due to severe physical or mental conditions, language barriers, or were institutionalized or hospitalized at the time of the survey were excluded from the study. The study commenced in February 2019 but was suspended during the lockdown period (April 2020 – July 2020) in Singapore in response to the Coronavirus pandemic. It was resumed in July 2020 while adhering to safe distancing and masking policies, and recruitment was closed in September 2020. In all, 2895 participants were recruited from the general population (screened 5698; response rate 66.2%; eligibility rate 76.8%). However, 28 participants who were recruited in the period of 1st April 2020 (the month in which lockdown measures against COVID first came into effect in Singapore) to 1st September 2020 were not included in analyses, and the remaining 2867comprised the study sample. Prior to the lockdown period, recreational and fitness facilities were still open, although social distancing had been recommended since March 2020. Written informed consent was obtained from all respondents prior to the survey, with parental consent being sought for those aged 18–20 years as the official age of majority in Singapore is 21 years and above. All study procedures were conducted in accordance with ethical guidelines (Domain Specific Review Board ref.: 2018/00430).

### Measures

#### Sociodemographic information

Respondents’ sociodemographic information such as age, sex, ethnicity, education, marital status, employment status, and monthly personal income were also collected. Self-reported weight and height were also obtained from respondents to calculate body mass index (BMI) scores. Based on World Health Organization [72] international cut-offs, respondents were classified into four groups: underweight (BMI < 18.5 kg/m^2^), normal range (≥ 18.5 kg/m^2^ and < 25 kg/m^2^), overweight (≥ 25 kg/m^2^ and < 30 kg/m^2^), and obese (≥ 30 kg/m^2^).

#### Physical activity and sedentary behaviour

The Global Physical Activity Questionnaire (GPAQ) is a 16-item instrument developed by the World Health Organization to measure physical activity [73]. Translations of the GPAQ to Mandarin, Malay, and Tamil were permitted by the publisher. Respondents were asked about the duration and frequency of vigorous and moderate- intensity activities for work, transport, or leisure during a typical week. The GPAQ has demonstrated fair-to-moderate correlations with moderate-to-vigorous physical activity measured by an accelerometer in Singapore in a prior study [17]. A cut-off to dichotomize physical activity was applied following 2020 World Health Organization guidelines [9]. Those who met the following criteria for physical activity for work, during transport and leisure time throughout the week were classified as “sufficiently active”: i) At least 150 min of moderate-intensity physical activity, OR ii) 75 min of vigorous-intensity physical activity OR iii) An equivalent combination of moderate- and vigorous-intensity physical activity. Individuals who did not meet the criteria were classified as “insufficiently active”.

The GPAQ also contains a single item: “How much time do you usually spend sitting or reclining on a typical day?”, which was used as a measure of sedentary behaviour. This item has demonstrated moderate correlation with accelerometery-based measures of sedentary behaviour [18]. Based on two meta-analyses [14, 42], ≥7-h/day cut-off was utilized to differentiate between levels of self-reported sedentary behaviour.

#### Chronic physical conditions and multimorbidity

The questionnaire used to capture chronic physical conditions had been previously employed in population-wide studies [1, 16] and was adapted from the World Mental Health Composite International Diagnostic Interview [39]. Respondents were asked whether a doctor had diagnosed them with any of the following 18 chronic conditions that were prevalent within the Singapore population: asthma, diabetes, hypertension or high blood pressure, arthritis or rheumatism, cancer, neurological condition, Parkinson’s disease, stroke or major paralysis, congestive heart failure, heart disease, back problems including disk or spine, stomach ulcer, chronic inflamed bowel disease or enteritis or colitis, thyroid disease, kidney failure, migraine headaches, chronic lung diseases, and hyperlipidaemia or high cholesterol. For each respondent, a total number was created by summing the number of endorsed chronic physical conditions. This variable was further categorized into the following groups: individuals with i) no chronic conditions, ii) one chronic condition, and iii) multimorbidity (i.e., two or more chronic conditions).

#### Health-related quality of life (HRQoL)

The Short Form Health Survey version 2 (SF-12v2) is a 12-item questionnaire designed to assess HRQoL amongst patient populations and replicates summary scores of the SF-36 [70]. This questionnaire has been validated in the Singapore population [44]. The 12-items cover eight sub-domains: general health, physical functioning, role physical, bodily pain, vitality, social functioning, role emotional, and mental health. Responses are weighted and summarized into two composite scores using a scoring algorithm provided by the developers: Physical Component Score (PCS) which measures physical HRQoL, and Mental Component Score (MCS) which measures mental HRQoL. Higher scores indicate better HRQoL. Internal consistency of the PCS and MCS were high, with Cronbach α values of 0.80 and 0.82 respectively.

### Statistical analysis

All analyses within the present study were conducted with Stata version 15, with survey weights utilized to adjust for age and ethnicity post-stratification, oversampling, and non-response. Both frequency and survey-weighted percentages were provided for descriptive statistics. Prevalence of those who were insufficiently active as well as those with sedentary behaviour of ≥7 h/day were determined. All regression analyses utilized survey weights to account for complex survey design. Firstly, a multivariable logistic regression was conducted to determine which sociodemographic variables (i.e., age, sex, ethnicity, education, marital status, monthly personal income, BMI, and chronic physical conditions) were associated with physical activity (insufficiently active vs sufficiently active). Descriptive statistics were presented to examine the percentage of time spent in each domain relative to the overall level of physical activity across sociodemographic variables that were identified to be significantly associated with physical activity. Next, a multivariable logistic regression model was utilized to estimate the association between sociodemographic variables and sedentary behaviour (≥7 h/day vs < 7 h/day). Lastly, two multivariable linear regressions models were utilized to examine whether physical activity and sedentary behaviour were associated with physical and mental health, while adjusting for the effect of sociodemographic correlates.

## Results

### Sociodemographic characteristics

The sociodemographic characteristics of the full sample (*N* = 2867) split by physical activity levels are presented in Table 1. Each age group was well represented in the sample, with most respondents in the 18 to 34-year-old age group and least in the 65 and above age group. There were approximately equal number of males and females within the sample. 53.6% of respondents had BMI in the normal range, while 7.0% were underweight, 26.3% were overweight, and 9.0% were obese. 26.3% of respondents had at least one chronic condition, while 27.1% had multimorbidity.

**Table 1:** Sociodemographic characteristics of overall sample and split by physical activity levels

Categorical Variables	Overall (Percentages displayed by Columns)		Physical Activity Levels^a^ (Percentages displayed by rows)				
			Insufficiently Active		Sufficiently Active		
	*N* = 2867		*n* = 458, weighted 16.6%		*n* = 2408, weighted 83.4%		*p*^*b*^
	n	Weighted %	n	Weighted %	n	Weighted %	
Age							0.003
18 to 34	814	29.9%	79	12.0%	735	88.0%	
35 to 49	711	28.2%	96	17.1%	614	82.9%	
50 to 64	766	26.7%	119	16.9%	647	83.1%	
65 and above	576	15.2%	164	24.2%	412	75.8%	
Sex							0.36
Female	1458	51.6%	266	17.6%	1191	82.4%	
Male	1409	48.4%	192	15.6%	1217	84.5%	
Ethnicity							< 0.001
Chinese	791	75.9%	141	17.7%	650	82.3%	
Malay	961	12.7%	140	12.7%	821	87.3%	
Indian	908	8.6%	156	14.7%	751	85.1%	
Others	207	2.9%	21	9.9%	186	90.1%	
Education							0.15
Primary and Below	631	20.4%	159	22.3%	472	77.7%	
Secondary School	681	20.3%	110	16.4%	571	83.6%	
Pre-U/Junior College	123	4.7%	12	6.9%	111	93.1%	
Vocational Institute/ITE	263	6.6%	30	11.4%	233	88.6%	
Diploma	474	18.5%	55	15.3%	419	84.7%	
Degree, professional certification, and above	695	29.6%	92	16.3%	602	83.7%	
Marital Status							0.65
Single	723	29.3%	82	14.1%	641	85.9%	
Married/Cohabiting	1840	61.6%	307	17.5%	1532	82.5%	
Divorced/Separated/Widowed	303	9.2%	69	18.4%	234	81.6%	
Refused^c^	1	0.0%	0	0.0%	1	100.0%	
Employment							0.85
Employed	1911	70.4%	257	16.3%	1653	83.7%	
Economically inactive	826	25.5%	180	17.9%	646	82.1%	
Unemployed	130	4.0%	21	13.6%	109	86.4%	
Monthly Personal Income (SGD)							0.98
No income/ Below $2000	1441	45.2%	272	15.5%	1168	84.5%	
$2000 - $3999	689	24.0%	91	19.2%	598	80.8%	
$4000 - $5999	317	12.9%	42	16.8%	275	83.2%	
$6000 - $9999	180	7.8%	26	16.0%	154	84.0%	
$10,000 and above	116	5.7%	16	17.7%	100	82.3%	
Don’t Know/Refused^c^	124	4.5%	11	12.7%	113	87.3%	
BMI							0.19
Underweight	150	7.0%	28	20.2%	122	79.8%	
Normal range	1253	53.6%	184	16.2%	1068	83.8%	
Overweight	848	26.3%	120	13.5%	728	86.5%	
Obese	415	9.0%	78	20.2%	337	79.8%	
Refused^c^	201	4.1%	48	27.8%	153	72.2%	
Chronic physical conditions							0.002
No chronic condition	1229	46.2%	153	16.0%	1076	84.1%	
One chronic condition	754	26.3%	95	11.6%	658	88.4%	
Multimorbidity	876	27.1%	208	22.7%	668	77.3%	
Missing^c^	8	0.3%	2	3.5%	6	96.5%	
Sedentary behaviour							< 0.001
< 7 h/day	1569	52.3%	183	12.4%	1385	87.5%	
≥ 7 h/day	1297	47.7%	275	21.1%	1022	78.9%	
Missing^c^	1	0.0%	0	0.0%	1	100.0%	
Continuous Variables	Overall		Insufficiently Active		Sufficiently Active		*p*^*b*^
	Mean	S.D.	Mean	S.D.	Mean	S.D.	
12-item Short Form Survey (SF-12)							
Physical Component Score	51.6	6.8	48.9	8.2	52.1	6.3	**< 0.001**
Mental Component Score	51.8	7.9	51.7	8.3	51.8	7.8	0.85

^a^ One case was removed from analyses as advised by GPAQ analysis guide, as their total time spent exercising exceeded 24 h/1440 min. 5.3% of study population reported having no physical activity

^b^Bivariate associations between categorical variables and physical activity was examined via chi-square analyses. Associations between physical activity and SF-12 scores was tested via t-tests

^c^Respondents who indicated Don’t Know/Refused and had missing data were not included in bivariate analyses and were excluded from subsequent regression analyses

### Prevalence of physical activity and sedentary behaviour

83.4% of the study population were sufficiently active (i.e., met WHO guidelines for physical activity), while 16.6% were insufficiently active. The median self-reported number of sedentary hours was 6 h (IQR: 4, 9). 47.7% reported that they had sedentary behaviour of ≥7 h/day. Sociodemographic characteristics of the sample split by sedentary behaviour can be found in Supplementary Table 1.

### Correlates of physical activity

Results of the logistic regression models examining the correlates of physical activity can be found in Table 2. Individuals aged 65 years and above were more likely to be insufficiently active (O.R.: 2.5, *p* = 0.01), as compared to those aged 18 to 34 years. Similarly, individuals who reported having a monthly personal income of SGD 2000 to 3999 were more likely to be insufficiently active (O.R.: 1.8, *p* = 0.01) than those with below SGD 2000 or no income. Those of Malay ethnicity (O.R.: 0.7, *p* = 0.02) and those with one chronic condition (O.R.: 0.6, *p* = 0.03) were less likely to have insufficient physical activity, as compared to those of Chinese ethnicity, and those with no chronic conditions, respectively.

**Table 2:** Results of logistic regression analyses examining correlates of physical activity and sedentary behaviour

	Physical Activity^a^ (Logistic Regression Model)				Sedentary Behaviour^b^ (Logistic Regression Model)			
	Insufficiently active vs sufficiently active				≥7 h vs < 7 h			
	O.R.	95% CI		*p*	O.R.	95% CI		*p*
		Lower	Upper			Lower	Upper	
Age								
18 to 34	ref				ref			
35 to 49	1.3	0.8	2.3	0.26	**0.7**	0.5	1.0	**0.04**
50 to 64	1.5	0.8	2.7	0.20	**0.6**	0.4	0.9	**0.01**
65 and above	**2.5**	1.3	4.9	**0.01**	**0.5**	0.3	0.9	**0.02**
Sex								
Female	ref				ref			
Male	0.9	0.6	1.2	0.40	0.8	0.7	1.1	0.22
Ethnicity								
Chinese	ref				ref			
Malay	**0.7**	0.5	0.9	**0.02**	0.8	0.6	1.1	0.14
Indian	0.9	0.7	1.2	0.42	0.8	0.6	1.0	0.06
Others	0.6	0.3	1.1	0.13	0.8	0.6	1.2	0.32
Education								
Degree, professional certification, and above	ref				ref			
Primary and Below	1.4	0.7	2.6	0.35	**0.6**	0.4	1.0	**0.04**
Secondary School	1.1	0.6	2.0	0.72	0.7	0.4	1.0	0.07
Pre-U/Junior College	0.5	0.2	1.3	0.15	0.6	0.3	1.1	0.08
Vocational Institute/ITE	0.9	0.4	1.9	0.75	0.6	0.4	1.1	0.10
Diploma	1.1	0.6	1.8	0.76	1.0	0.6	1.4	0.82
Marital Status								
Married/Cohabiting	ref				ref			
Single	1.2	0.7	1.9	0.53	**2.1**	1.5	2.9	**< 0.001**
Divorced/Separated/Widowed	0.9	0.5	1.6	0.80	1.0	0.7	1.7	0.85
Employment								
Employed	ref				ref			
Economically inactive	1.1	0.7	1.6	0.81	0.9	0.7	1.3	0.66
Unemployed	0.9	0.4	2.1	0.87	0.6	0.3	1.1	0.09
Monthly Personal Income (SGD)								
No income/ Below $2000	ref				ref			
$2000 - $3999	**1.8**	1.2	2.8	**0.01**	**1.9**	1.3	2.6	**0.001**
$4000 - $5999	1.7	0.9	3.1	0.08	**1.9**	1.2	3.0	**0.01**
$6000 - $9999	1.4	0.7	3.0	0.33	**2.6**	1.4	4.7	**0.001**
$10,000 and above	1.8	0.8	4.3	0.16	**4.4**	2.2	8.7	**< 0.001**
Body Mass Index								
Normal range	ref				ref			
Underweight	1.7	0.9	3.0	0.11	1.1	0.6	1.9	0.73
Overweight	0.8	0.5	1.2	0.26	1.3	1.0	1.7	0.07
Obese	1.5	0.9	2.5	0.14	**2.3**	1.6	3.4	**< 0.001**
Chronic physical conditions								
No chronic condition	ref				ref			
One chronic condition	**0.6**	0.4	0.9	**0.03**	1.0	0.7	1.3	0.76
Multimorbidity	1.2	0.8	1.7	0.45	**1.6**	**1.1**	**2.2**	**0.01**

O.R. – Odds Ratio; 95% CI – 95% confidence interval of OR

Bold print highlights statistically significant O.R

Analyses utilized survey weights to account for complex survey design

^a^After accounting for listwise deletion of missing data, cases in logistic regression model: 2544. Insufficiently active: 16.4% (*n* = 399); Sufficiently active (reference group): 83.6% (*n* = 2145)

^b^After accounting for listwise deletion of missing data, cases in logistic regression model: 2544. ≥7 h: 48.2% (*n* = 1178); < 7 h (reference group): 51.8% (*n* = 1366)

### Description of physical activity domains across age, ethnicity, income, and chronic conditions

**Fig. 1:**
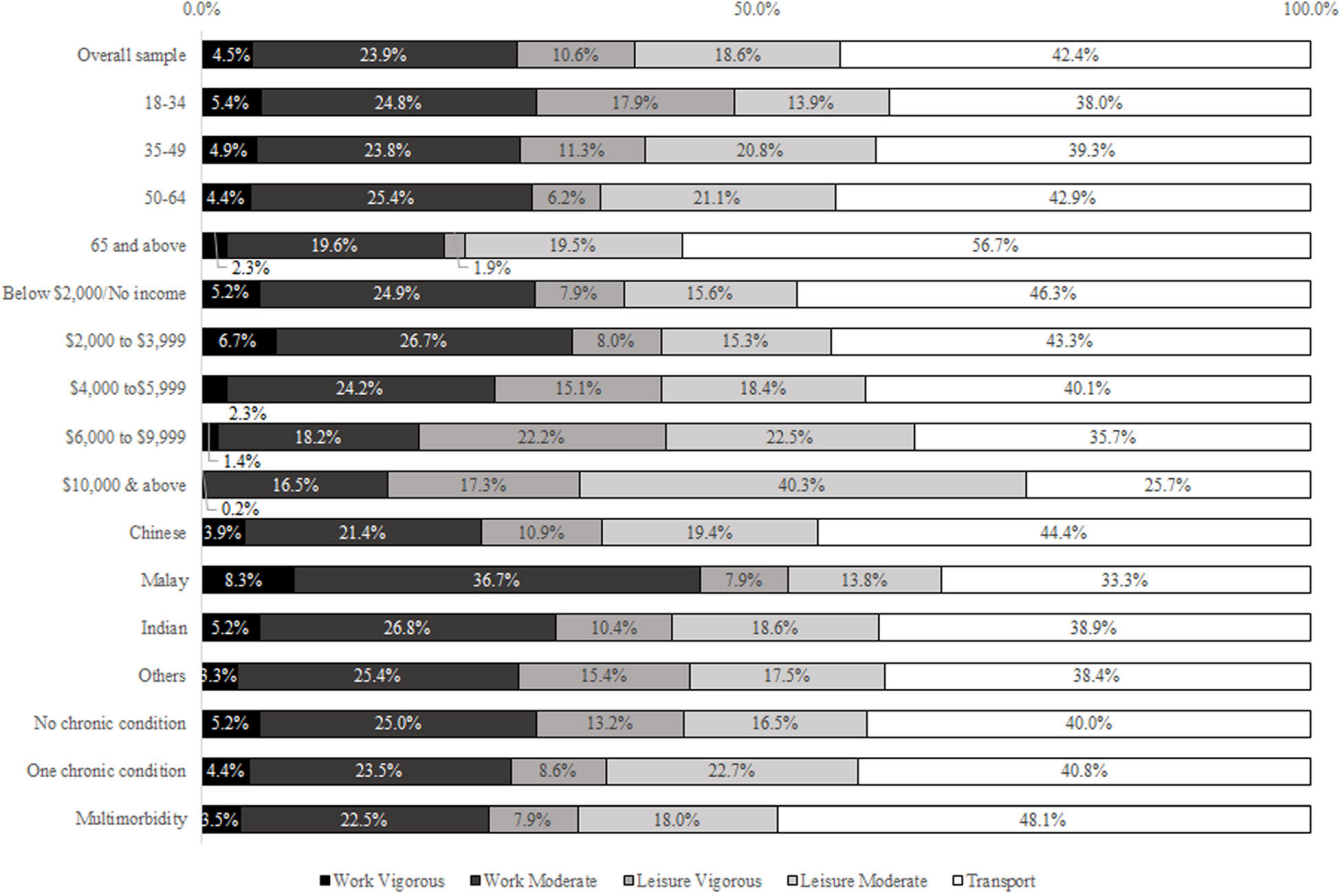
Contribution of specific domains and intensities to the amount of minutes spent per week in physical activity by income, ethnicity, and chronic conditions, amongst those who reported having any physical activity (*n* = 2716, weighted 94.7%)

The percentages of time spent per week in the different physical activity domains by age group, income, ethnicity, and chronic conditions, amongst those who reported having any amount of physical activity (*n* = 2716, 94.7%) are shown in Fig. 1. These variables were selected as they were identified as significant correlates of physical activity in the earlier logistic regression model. In general, older adults appear to spend less percentage of their time in work-related physical activity than their younger counterparts. Older adults also spent a lesser percentage of their time (1.9%) in vigorous leisure-related physical activity than those aged 18 to 34 (17.9%). Those with higher incomes spent less percentage of their time in work-related physical activity, and increasingly more time in leisure-related physical activity, than their counterparts with lower income. Those of Malay ethnicity spent a smaller percentage of their time (21.7%) in both moderate and vigorous leisure activities in comparison to Chinese (30.3%). Individuals with one chronic condition spent a larger percentage of their time in leisure activities (31.3%) than those with no chronic conditions (29.8%) and those with multimorbidity (26.0%).

### Correlates of sedentary behaviour

The logistic regression model examining correlates of sedentary behaviour can be found in Table 2. Age was negatively associated with sedentary behaviour, with likelihood of having ≥7 h/day of sedentary behaviour decreasing as age increases. Those aged 35 to 49 years (O.R.: 0.7, *p* = 0.04), 50 to 64 (O.R.: 0.6, *p* = 0.01), 65 years and above (O.R.: 0.5, *p* = 0.02), had less sedentary behaviour than those aged 18 to 34 years. Those with an education of primary school and below had lower likelihoods of having sedentary behaviour than those who had attained a degree (O.R.: 0.6, *p* = 0.04). In contrast, individuals who were single were more likely to have sedentary behaviour than those who were married or cohabiting (O.R.: 2.1, *p* <  0.001). Similarly, higher income groups were associated with higher likelihoods of endorsing sedentary behaviour. Individuals with monthly incomes of SGD 2000 to 3999 (O.R.: 1.9, *p* = 0.001), 4000 to 5999 (O.R.: 1.9, *p* = 0.01), 6000 to 9999 (O.R.: 2.6, *p =* 0.001) and 10,000 and above (O.R.: 4.4, *p* <  0.001) were more likely to have ≥7 h of sedentary behaviour than those with incomes below SGD 2000 or no income. Obesity (O.R.: 2.3, *p* <  0.001) and multimorbidity (O.R.: 1.6, *p* = 0.01) were also associated with increased likelihood of having sedentary behaviour.

### Association of Physical Activity and Sedentary Behaviour with HRQoL

Results of the linear regression analyses examining the association between physical activity and sedentary behaviour with physical and mental HRQoL while adjusting for the effect of sociodemographic correlates can be found in Table 3. Insufficient physical activity was significantly associated with poorer physical HRQoL (B: − 2.5, *p* <  0.001). Sedentary behaviour was not significantly associated with physical or mental HRQoL.

**Table 3:** Results of linear regression analyses examining association between physical activity, sedentary behaviour with physical and mental HRQoL

	Physical Component Score (Physical HRQoL) ^a^				Mental Component Score (Mental HRQoL) ^a^			
	B	95% CI		*p*	B	95% CI		*p*
		Lower	Upper			Lower	Upper	
Physical activity								
Sufficiently Active	ref				ref			
Insufficiently Active	**−2.5**	−3.5	−1.4	**< 0.001**	−0.2	−1.6	0.9	0.71
Sedentary behavior								
< 7 h/day	ref				Ref			
≥ 7 h/day	−0.2	−0.9	0.6	0.60	−0.4	−1.4	0.5	0.41

B – unstandardized regression coefficient; 95% confidence interval of B

Bold print highlights statistically significant B

After accounting for listwise deletion of missing data, cases in each regression model: *n* = 2543

^a^Estimates are adjusted for sociodemographic correlates (i.e., age, sex, ethnicity, education, marital status, employment, income, BMI, chronic physical conditions)

## Discussion

### Prevalence and sociodemographic correlates of physical activity

The percentage of the study population who met recommendations for sufficient physical activity (83.4%), within this study was similar to that reported in 2017 and 2019 at approximately 80% (Health Promotion [31]). However, this indicates that the remaining 16.6% require further encouragement or assistance to meet the minimal physical activity requirements. It is to note that the cut-off applied in this study indicates the minimum recommended amount of at least 150 min of moderate physical activity, or 75 min of vigorous physical activity a week, and not the ideal amount of PA. The current WHO guidelines recommends that beyond 300 min of moderate physical activity a week or 150 min of vigorous physical activity is associated with additional health benefits [74].

The study highlights important age and ethnic differences in physical activity. Older adults aged 65 years and above had higher odds of having insufficient physical activity as compared to those aged 18 to 34 years. Older adults also appeared to spend less percentage of their time in vigorous physical activity. This is supported by research demonstrating a decline in physical activity with increasing age [11, 54]. Other studies have suggested that physiological systems tend to decline in older age, and many older adults suffer from frailty and mobility issues [8, 20, 58]. This may prevent them from participating in vigorous-intensity leisure activities and they may also be less likely to have occupations that involve physical activity. Though Singapore has employed a number of active aging programmes [50], more outreach, particularly in the relevant local dialects, is required to tie physical exercise in with active aging and emphasize the importance and relevance of exercise.

The study also revealed that those of Malay ethnicity were less likely to have insufficient physical activity than Chinese. This seems to have occurred as the Chinese ethnic group spent a smaller percentage of their time in work-related moderate to vigorous physical activity (MVPA). It is important to note that the Malay ethnic group spent a lower percentage of time in leisure-time MVPA (Chinese 30.3% vs Malay 21.7%). Recent studies have similarly reported that Singaporean Malays had lower leisure-time physical activity than their Chinese peers [24, 52]. Future initiatives could be implemented to promote overall physical activity across all ethnic groups, while providing extra encouragement for leisure time physical activity for Malays. For example, Müller-Riemenschneider et al. [52] also explored differences in types of exercise across ethnic groups, and found that the beneficial effect of running was consistent across multiple health outcomes for those of Malay ethnicity. Policymakers should work with relevant community and grassroots groups to engage and design culturally relevant programmes such as mosque-based exercise interventions, which have appeared to be effective in encouraging physical activity and quality of life [4].

Although many studies also report an association between income and physical activity, the direction of association appears to be in contrast with that of the present study. In general, extant literature reports that physical activity levels are positively related to income [19, 37, 57]. However, the present study found that those with personal incomes of SGD 2000 to 3999 were more likely to be insufficiently active than those earning less than SGD 2000. Previous research in the Singapore population has also reported that the prevalence of physical activity was highest in participants with monthly household income less than SGD 2000, with the prevalence of physical activity decreasing with higher income [71]. The reason for this occurrence is unclear, but the findings do highlight a specific group within the population that requires further examination. Descriptive statistics also indicate that those in the lower- income groups spent a smaller percentage of their time in leisure-time physical activity than higher income groups, possibly due to poorer access to facilities, financial or time constraints. Future studies should explore how occupation and leisure time MVPA contribute differently to health outcomes. In terms of recommendations, public health officials may consider ensuring equal access to recreational facilities and lowering of costs or providing subsidies for sports programmes and equipment. For example, given the health benefits of cycling [28] and the potential cost-effectiveness of building cycling networks [43], health officials may consider further development of cycling networks, providing subsidies for the purchase of bicycles, and proliferation of bicycle- sharing schemes. In a qualitative study conducted in 2017, limitations brought up by Singaporeans were a lack of cycling infrastructure, parking facilities, and safety concerns [59]. Local news has already reported a rise of cycling as a leisure time activity due to the COVID-19 pandemic as it allowed outdoor activity while being able to maintain safe distancing (Channel [12]), and further improvements to the cycling network may be able to further promote this leisure activity.

Interestingly, those with one chronic physical condition were less likely to have insufficient physical activity. It is possible that the Singapore government’s health campaigns such as the “War on Diabetes” and increased awareness of the impact of chronic physical conditions and the risk of multimorbidity could have prompted this group to exercise more regularly. It is also plausible that after being diagnosed with a chronic physical condition, they would be more health- conscious and therefore exercise regularly as prevention against further deterioration of their health.

### Sedentary behaviour and its sociodemographic correlates

A large proportion (47.7%) of the population appears to have sedentary levels of ≥7 h/day. If a threshold of ≥8 h/day was utilized, the present study reports a prevalence of 42.0%, which is still a substantial absolute increase in comparison with data collected in 2013 (37%) by Win et al. [71]. The prevalence of sedentary behaviour was highest amongst the 18- to 34-year-old age group (59.8%), with the likelihood of having ≥7 h/day of sedentary time decreasing as age increased. This was similarly reported in the Singapore population by Win et al. [71], who suggested that young/middle- aged adults tend to have more sedentary occupations. In contrast, being single, having a higher income and education level, obesity, and having two or more chronic physical conditions were associated with a greater likelihood of having sedentary behaviour. Extant literature has shown that single individuals were more likely to have higher sedentary behaviour [36, 67, 68, 71]. It is possible that married individuals may have less sedentary behaviour due to child- rearing practices or encouragement from their significant other to have a healthier lifestyle. Socioeconomic status has long been shown to be the most consistent factor that is positively related with sedentary behaviour across both Western [41, 62] and Asian cultures [15, 22, 51, 71]. This is likely because individuals with higher income and educational levels are more likely to work in sedentary occupations. Müller et al. [51] also suggested that increased income may be related to the purchase of items such as cars and home entertainment that may encourage sedentary behaviour. The bulk of research has provided evidence that breaking up prolonged and uninterrupted periods of sedentary time may provide beneficial metabolic effects [5, 32, 47]. Therefore, there is a need for more campaigns and interventions to reduce sedentary behaviour in the workplace, and amongst those with higher incomes. In this vein, randomized control trials utilizing height- adjustable workstations have found a reduction in time spent sitting and improvements in mental health benefits and job performance [13, 23, 53]. However, no such intervention has been assessed within Singapore. Public health officials should aim to implement and evaluate the practice of height-adjustable workstations and behavioural change strategies.

The positive relationship between obesity and sedentary behaviour has been corroborated by existing literature. For example, greater sedentary time is associated with higher BMIs and increased waist circumference [22, 33]. In a nationwide and longitudinal study of women in the United Kingdom, Hu et al. [34] reported that every 2 h per day increase in television watching or sitting at work was associated with a 23 and 5% increase risk of obesity, respectively. The present study reported that 26.3% of respondents were overweight, and 9% were obese. Therefore, there is an urgent requirement for a reduction in sedentary behaviour amongst obese individuals to ensure their health and welfare.

The present study also found a significant positive association between multimorbidity and sedentary behaviour that is supported by existing research. For example, George et al. [26] found that individuals who had higher sedentary behaviour were more likely to report having any chronic condition. Furthermore, Katzmarzyk et al. [38] found a dose-response association between sitting time and mortality from all causes and CVD that is independent of physical activity. It is also plausible that individuals with multimorbidity are more sedentary because of mobility and issues with activities of daily living. For example, Lau et al. [45] examined a sample of patients with multimorbidity in Singapore and reported that sedentary behaviour of ≥7 h/day was positively associated with problems with mobility, self-care, and usual activities. The debilitating nature of multimorbidity may have prevented these individuals from adopting or even initiating an active lifestyle. This finding highlights a need for further resource allocation for this group, who might be unable to break up sedentary bouts or engage in physical activity.

### Physical activity, sedentary behaviour and physical and mental HRQoL

Physical activity was associated with lower physical HRQoL and this finding is corroborated by existing literature expounding the benefits of physical activity [6, 40, 55]. There might be several explanations for this association such as improved self-esteem in which positive perceptions of competency and improved self-worth from exercise may lead to better perceptions of health [60]. It is unclear why physical activity was not associated with mental HRQoL, since studies have reported its beneficial effects for mental well-being and negative association with mental illness [25, 27]. Furthermore, a nation-wide study in Singapore found that physical activity expenditure was positively associated with PCS and MCS of the SF-12 [46]. In contrast, findings on the association between sedentary behaviour and HRQoL have been mixed, with some studies reporting no association [7, 48, 66], and others suggesting a negative association [30, 65], and that sedentary behaviours were associated with increased risk of depression [35, 75] and anxiety [2].

### Limitations and avenues for future research

The sample for the present study was representative of the resident population of Singapore, and the results are therefore generalizable. However, the present study does have some limitations. Firstly, levels of physical activity and sedentary behaviour may have been affected by the pandemic. The present study has sought to mitigate this confounding factor to a reasonable degree as the study population only included individuals recruited prior to the lockdown period in April 2020. Prior to this, sports facilities were still open, and heavy restrictions on social activities and remote work had yet to be implemented; thus patterns of physical activity and sedentary behaviour may not have been affected to a large extent. However, due to the unforeseen nature of the pandemic, it is plausible that some confounding may have occurred. For example, upon receiving news of the pandemic in December 2019, individuals may have increased their exercise to improve their health or may have avoided physical activity in public spaces and/or sports facilities to reduce contagion. Unfortunately, the present study was also not able to conduct comparative analyses of physical activity during the pandemic as recruitment was halted during the period of April to July 2020 for the health and safety of both staff and potential respondents, and the number of respondents recruited after the restrictions were lifted in July was too small (*n* = 28). Secondly, this study is cross-sectional in nature and is thus unable to determine causal relationships between variables. Third, sedentary behaviour and physical activity were both self-reported and were subject to recall bias. A recent systematic review reported that single-item self-report measures, as is the case of sedentary behaviour in the present study, generally underestimate sedentary time compared to device measures [56]. Similarly some studies reported that individuals tend to overestimate their levels of physical activity [61, 69]. The measurement of sedentary behaviour in the present study did not account for the number of breaks nor the length of a sedentary bout. Furthermore, the GPAQ does not assess sedentary behaviour across different types of activities (e.g., TV, gaming) or domains (e.g., occupational, leisure, transport). Future studies are therefore encouraged to utilize accelerometers as an objective measure to examine both physical activity and sedentary behaviour.

## Conclusion

The present study highlights that although most Singaporeans met guidelines for MVPA, 16.6% of the population had insufficient physical activity. Furthermore, about half of the population appeared to spend ≥7 h/day sedentary. As expected, insufficient physical activity was associated with poorer physical HRQoL. Policymakers should allocate more resources to promoting moderate physical activity and encouraging the breaking up of prolonged sedentary periods for middle- and high-income groups, especially during working hours. For those in the lower- income groups, it would be prudent to encourage more leisure-time exercise.

## Supplementary Information

**Additional file 1.**

## Data Availability

The datasets used and/or analysed during the current study are available from the corresponding author on reasonable request.

## Abbreviations

GPAQ: Global Physical Activity Questionnaire
HRQoL: Health-related Quality of Life
MVPA: Moderate to Vigorous Physical Activity
OR: Odds Ratio
SF12: 12-item Short Form Survey
SGD: Singapore Dollars
WHO: World Health Organization
