# Supplementary material for: Prevalence and patterns of physical activity, sedentary behaviour, and their association with health-related quality of life within a multi-ethnic Asian population
Source: BMC Public Health. 2021 Oct 25;21:1939. doi: 10.1186/s12889-021-11902-6 (PMC8544627; doi:10.1186/s12889-021-11902-6)
Supplement: Supplementary file 1 — Additional file 1. [file 12889_2021_11902_MOESM1_ESM.docx]

**Appendix**

| Supplementary Table 1 | | | | | | | |
| --- | --- | --- | --- | --- | --- | --- | --- |
| Sociodemographic characteristics of the sample split by sedentary behaviour | | | | | | | |
|  | Overall  (Percentages displayed by Columns) | | Sedentary Behaviour^a^  (Percentages displayed by rows) | | | |  |
|  |  |  | <7hrs/day | | ≥7hrs/day | |  |
|  | N = 2867 | | n=1569, weighted 52.3% | | n=1297, weighted 47.7% | |  |
|  | n | Weighted % | n | Weighted % | n | Weighted % | *p^a^* |
|  |  |  |  |  |  |  |  |
| Age |  |  |  |  |  |  |  |
| 18 to 34 | 814 | 29.9% | 372 | 40.2% | 442 | 59.8% | **< 0.001** |
| 35 to 49 | 711 | 28.2% | 369 | 45.5% | 342 | 54.5% |  |
| 50 to 64 | 766 | 26.7% | 476 | 62.0% | 290 | 38.0% |  |
| 65 and above | 576 | 15.2% | 352 | 71.7% | 223 | 28.3% |  |
| Sex |  |  |  |  |  |  |  |
| Female | 1458 | 51.6% | 813 | 52.9% | 645 | 47.1% | 0.62 |
| Male | 1409 | 48.4% | 756 | 51.6% | 652 | 48.4% |  |
| Ethnicity |  |  |  |  |  |  |  |
| Chinese | 791 | 75.9% | 416 | 51.5% | 375 | 48.5% | **0.02** |
| Malay | 961 | 12.7% | 554 | 57.0% | 407 | 43.1% |  |
| Indian | 908 | 8.6% | 495 | 54.1% | 412 | 45.8% |  |
| Others | 207 | 2.9% | 104 | 46.9% | 103 | 53.1% |  |
| Education |  |  |  |  |  |  |  |
| Primary and Below | 631 | 20.4% | 408 | 71.6% | 223 | 28.4% | **< 0.001** |
| Secondary School | 681 | 20.3% | 400 | 60.9% | 280 | 39.0% |  |
| Pre-U/Junior College | 123 | 4.7% | 69 | 60.4% | 54 | 39.6% |  |
| Vocational Institute/ITE | 263 | 6.6% | 154 | 53.8% | 109 | 46.2% |  |
| Diploma | 474 | 18.5% | 241 | 42.4% | 233 | 57.6% |  |
| Degree, professional certification, and above | 695 | 29.6% | 297 | 37.6% | 398 | 62.4% |  |
| Marital Status |  |  |  |  |  |  |  |
| Single | 723 | 29.3% | 322 | 38.7% | 401 | 61.4% | **< 0.001** |
| Married/Cohabiting | 1840 | 61.6% | 1079 | 57.0% | 761 | 43.0% |  |
| Divorced/Separated/Widowed | 303 | 9.2% | 168 | 64.4% | 134 | 35.6% |  |
| Refused^b^ | 1 | 0.0% | 0 | 0.0% | 1 | 100.0% |  |
| Employment |  |  |  |  |  |  |  |
| Employed | 1911 | 70.4% | 983 | 47.2% | 927 | 52.8% | **< 0.001** |
| Economically inactive | 826 | 25.5% | 506 | 64.2% | 320 | 35.8% |  |
| Unemployed | 130 | 4.0% | 80 | 65.8% | 50 | 34.2% |  |
| Monthly Personal Income (SGD) |  |  |  |  |  |  |  |
| No income/ Below $2,000 | 1441 | 45.2% | 897 | 64.8% | 543 | 35.2% | **< 0.001** |
| $2,000 - $3,999 | 689 | 24.0% | 345 | 44.9% | 344 | 55.1% |  |
| $4,000 - $5,999 | 317 | 12.9% | 151 | 44.3% | 166 | 55.7% |  |
| $6,000 - $9,999 | 180 | 7.8% | 63 | 35.7% | 117 | 64.3% |  |
| $10,000 and above | 116 | 5.7% | 41 | 28.4% | 75 | 71.6% |  |
| Don’t Know/Refused^b^ | 124 | 4.5% | 72 | 47.9% | 52 | 52.1% |  |
| BMI |  |  |  |  |  |  |  |
| Underweight | 150 | 7.0% | 79 | 45.5% | 71 | 54.5% | **0.01** |
| Normal | 1253 | 53.6% | 710 | 55.0% | 543 | 45.0% |  |
| Overweight | 848 | 26.3% | 454 | 50.3% | 393 | 49.7% |  |
| Obese | 415 | 9.0% | 194 | 40.2% | 221 | 59.8% |  |
| Refused^b^ | 201 | 4.1% | 132 | 67.4% | 69 | 32.6% |  |
| Chronic physical conditions |  |  |  |  |  |  |  |
| No chronic condition | 1229 | 46.2% | 676 | 50.1% | 553 | 49.9% | 0.78 |
| One chronic condition | 754 | 26.3% | 400 | 54.4% | 354 | 45.6% |  |
| Multimorbidity | 876 | 27.1% | 488 | 54.0% | 387 | 46.0% |  |
| Missing^b^ | 8 | 0.3% | 5 | 42.2% | 3 | 57.8% |  |
| Physical Activity |  |  |  |  |  |  |  |
| Insufficiently Active | 1569 | 52.3% | 183 | 39.2% | 275 | 60.8% | **< 0.001** |
| Sufficiently Active | 1297 | 47.7% | 1385 | 54.9% | 1022 | 45.1% |  |
| Excluded^b^ | 1 | 0.0% | 1 | 100.0% | 0 | 0.0% |  |
|  |  |  |  | |  | |  |
|  | Overall | | <7hrs/day | | ≥7hrs/day | |  |
| Continuous Variables | Mean | S.E. | Mean | S.D. | Mean | S.D. | *p^a^* |
| 12-item Short Form Survey (SF-12) |  |  |  |  |  |  |  |
| Physical Component Score | 51.6 | 6.8 | 51.2 | 6.6 | 52.0 | 6.9 | **0.02** |
| Mental Component Score | 51.8 | 7.9 | 52.5 | 7.7 | 51.0 | 8.0 | **< 0.001** |
| ^a^Bivariate associations between categorical variables and physical activity was examined via chi-square analyses. Associations between physical activity and SF-12 scores was tested via t-tests.  ^b^Respondents who indicated Don’t Know/Refused and had missing data were not included in bivariate analyses and were excluded from subsequent regression analyses | | | | | | | |
